# Role of microRNA-30c Targeting ADAM19 in Colorectal Cancer

**DOI:** 10.1371/journal.pone.0120698

**Published:** 2015-03-23

**Authors:** Qian Zhang, Lei Yu, Dandan Qin, Rui Huang, Xiaochen Jiang, Chendan Zou, Qingchao Tang, Yinggang Chen, Guiyu Wang, Xishan Wang, Xu Gao

**Affiliations:** 1 Department of Colorectal Surgery, the Second Affiliated Hospital of Harbin Medical University, Harbin, Heilongjiang, 150086, PR China; 2 Colorectal Cancer Institute of Harbin Medical University, Harbin, Heilongjiang, 150086, PR China; 3 Department of Nephrology, the Second Affiliated Hospital of Harbin Medical University, Harbin, Heilongjiang, 150086, PR China; 4 Department of Biochemistry and Molecular Biology, Harbin Medical University, Harbin, China; University of Saarland Medical School, GERMANY

## Abstract

MicroRNAs (miRNAs) are deregulated in a number of cancers including colorectal cancer. MiR-30c belongs to miR-30 family, and is involved in a variety of malignant diseases. In this study, we detected the expression of miR-30c in colon cancer cell lines and clinical colon cancer specimens. MiR-30c was shown to be dramatically down-regulated both in cell lines and cancer tissues. Additionally, miR-30c could inhibit cancer cell growth, migration and invasion in vitro. Consistently, stable over-expression of miR-30c inhibited the growth and lung metastasis of colon cancer cell xenografts in vivo. Furthermore, bioinformatics algorithm and luciferase reporter assay indicated ADAM19 as a direct target of miR-30c. Of interest, further experiments demonstrated that inhibition of ADAM19 by miR-30c partially mediated the anti-tumor effect of miR-30c. Overall, our study provides the new insight that miR-30c inhibited colon cancer cells via targeting ADAM19. Thus, miR-30c might serve as a promising therapeutic strategy for colon cancer treatment.

## Introduction

MicroRNAs (miRNAs) are highly conserved, 22 nucleotides long non-coding RNAs. MiRNA could regulate gene expression post-transcriptionally by interacting with complementary sequences (usually located in the 3’-untranslated region (3’-UTR) of nucleotide) of messenger RNA (mRNA) targets. These interactions may lead to the protein translation inhibition or target mRNAs degradation depending on whether miRNAs and their targets are perfectly complementary [1]. MiRNAs are dysregulated in a variety of cancers and miRNAs play an important role in tumorigenesis [2,3,4,5]. A number of miRNAs have been found to act as tumor suppressors [2,6,7,8]. In recent years, miRNAs have been recognized as critical regulators in development and progression of cancer including colorectal cancer (CRC) [9,10,11,12].

CRC is the third most common cancer in males and females, with an estimated 142820 new cases and 50830 deaths in the United States in 2013 [13]. Although substantial progress has been made in the past decades, including surgical treatment, radiotherapy and chemotherapy, the survival rate of CRC patients has changed little. It is of vital importance to search early detection method due to increased metastasis and mortality of advanced high-grade CRC.

MiR-30c is a member of the miR-30 family. Five distinct mature miRNA sequences are included in this family: miR-30a/miR-30c-2, miR-30d/miR-30b and miR-30e/miR-30c-1 [14]. Accumulating evidences indicate that the deregulation of miR-30c contributes to various malignant tumors, including breast cancer, endometrial cancer, lung cancer, liver cancer [8,15,16,17]. MiR-30 suppresses the tumorous processes in these above cancers by directly interacting with their corresponding targets. Nevertheless, there are relatively few studies available reporting the implication of miR-30c in the progression of colon cancer.

In our study, we determined the potential effect of miR-30c on colon cancer progression. Our data indicated that miR-30c has lower expression level in human colon cancer samples. What’s more, we investigated the mechanisms underlying the role of miR-30c in colon cancer development. The results showed that miR-30c plays a crucial role in a plethora of biological processes via regulation ADAM19 in human colon cancer. Therefore, re-expressing miR-30c and/or interfering with ADAM19 function might be a promising colon cancer therapeutic strategy.

## Materials and Methods

### Clinical samples

Sixty colon cancer samples and their adjacent normal tissues were collected from colon cancer patients in the Second Affiliated Hospital of Harbin Medical University (Harbin, China) during surgery and immediately stored in liquid nitrogen until use. No patients had been treated with radiotherapy or chemotherapy before surgery. The study was approved by the Ethics Committee of the 2^nd^ Affiliated Hospital of Ha’erbin Medical University. All clinical investigation was conducted according to the principles expressed in the Declaration of Helsinki. The written informed consent was obtained from each patient and approved by the local ethics committee.

### Cell culture

HCT116 and SW620 human colon cancer cell lines were gifts from Pro. LQ[18] and were grown in RPMI1640 or L15 medium (GIBCO Laboratories, Grand Island, NY, USA) and HEK293T cells were grown in DMEM medium. All medium were supplemented with 10% fetal bovine serum, 100 U/ml penicillin G and 100 μg/ml streptomycin (GIBCO Laboratories, Grand Island, NY, USA). All cells were cultured at 37°C in a humidified incubator containing 5% CO2.

### Vectors construction, oligonucleotide synthesis and transfection

The hsa-miR-30c mimic, miR-30c inhibitor, mimic negative control (NC mimic), inhibitor negative control (NC inhibitor) sequences and human ADAM19 siRNA were from the Gene Pharma Company (Shanghai, China). Lipofectamine 2000 Transfection Reagent (Invitrogen, Carlsbad, CA, USA) was used to transfect cells. ADAM19 cDNA without its 3’-UTR (2757 bp) were inserted into pcDNA3.1(+) (Invitrogen, Carlsbad, CA, USA) to build the plasmid pcDNA3.1(+)-ADAM19. A 513-bp segment containing pre-miR-30c was ligated to the pcDNA3.1(+) vector to construct stable miR-30c overexpression cells. Table 1 listed all related DNA sequences.

**Table 1:** Primers for plasmid construction, qRT-PCR, and oligonucleotide.

Name		sequence
**Plasmid construction**		
pcDNA3.1(+) miR-30c	F	GCAGGATCCACTAAGTAAGTGTCATATAGCC
	R	GCCTCGAGCAAGTTAACCAGAACTTTTGTC
pcDNA3.1(+)-ADAM19	F	CGCGGATCCACCGCCGGGCAGT
	R	CTTCTCGAGGGTTCCATGGCCATGGGT
ADAM19 3’UTR WT	F	GCCAACTAGTAAGGTCCCCTTCCCCAG
	R	CTAACAAGCTTAAAAGGCACCATGGCCCATTC
ADAM19 3’UTR MUT	F	GCCAACTAGTAAGGTCCCCTTCCCCAG
	R	GGTGGACAAGCTTGAAGAAGATCTGTTTTCCG
**qPCR**		
miR-30c stem loop		GTCGTATCCAGTGCGTGTCGTGGAGTCGGCAATTGCACTGGATACGACAGCTGAG
miR-30c	F	GGGGTGTAAACATCCTACACTC
	R	ATTGCGTGTCGTGGAGTCG
ADAM19	F	CACTCCGAGAATGCCATTGG
	R	TCCCTCCTGTTGCATCCATT
Actin	F	TACCTCATGAAGATCCTCACC
	R	TTTCGTGGATGCCACAGGAC
snRNAU6	F	GCTTCGGCAGCACATATACTAAAAT
	R	CGCTTCACGAATTTGCGTGTCAT
E-cadherin	F	TGCCCAGAAAATGAAAAAGG
	R	GTGTATGTGGCAATGCGTTC
N-cadherin	F	ACAGTGGCCACCTACAAAGG
	R	CCGAGATGGGGTTGATAATG
Vimentin	F	GAGAACTTTGCCGTTGAAGC
	R	GCTTCCTGTAGGTGGCAATC
**Oligonucleotide**		
miR-30c mimic		UGUAAACAUCCUACACUCUCAGC UGAGAGUGUAGGAUGUUUACAUU
Negative Control (NC)		UUCUCCGAACGUGUCACGUTT ACGUGACACGUUCGGAGAATT
miR-30c Inhibitor		GCUGAGAGUGUAGGAUGUUUACA
Inhibitor NC		CAGUACUUUUGUGUAGUACAA
siADAM19		UGGAUUUGUACCAUUCUUCTT GAAGAAUGGUACAAAUCCATT
Negative Control		AGACUGGAGACCUGCUAUCTT GUCAAUGCAAACGUGGAGUTT

### Stable transfection of miR-30c

2×10^5^ HCT116 cells were grown in a 60-mm plate until the confluence reached 60–70% in RPMI1640 media. The pcDNA3.1(+)-pre-miR-30c plasmid were then transfected into cells with Lipofectamine 2000 (Invitrogen, Carlsbad, CA). Stable cell lines were screened with 1mg/ml G418 (Sigma, Shanghai, China), and positive clones were identified using qRT-PCR.

### Quantitative real-time PCR (qRT-PCR)

Quantitative real-time RT-PCR was conducted as described previously [18]. Total RNA was isolated using TRIzol reagents (Invitrogen, Carlsbad, CA) according to the manufacturer’s instructions. Afterwards, RNA was reverse transcribed, subsequently the qRT-PCR was performed using a 7500 Real Time PCR System (Applied Biosystems, Mannheim, Germany) as follows: 94°C for 3 min and then 40 cycles of 94°C for 30s, 60°C for 30s, 72°C for 30s and one step of 82°C for 5s. Fluorescence was detected at the end of each cycle.

### Cell proliferation and colony formation assay

The 3-(4, 5-dimethylthiazol-2-yl)-2, 5-diphenyltetrazolium bromide (MTT) assay was adopted to assess cell viability as described previously [18]. To evaluate the colony formation ability, HCT116 or SW620 cells was seeded in 3.5-cm plates (1000 cells/dish). The colonies were fixed in methanol, stained with 0.1% crystal violet (Sigma, St Louis, MO, USA) and counted after 2 weeks.

### Migration and invasion assay

To determine the migration ability of cells in vitro, the Transwell assay was adopted as described previously [18]. To detect the invasion ability of cells, the membrane of the Transwell unit was coated with 40μl matrigel (BD Biosciences, SanJose, CA, USA) at 37°C for 4h to develop a reconstructed basement membrane. The cells were treated in the same way as migration assay. Wound healing assay was also adopted to investigate cell migration ability. Cells were seeded in 3.5-cm plates to a density of 70–80%. Afterwards, cells were scratched by 200μl pipette tips to construct an artificial wound. The migrating distance was measured after 24–72h.

### Western blot analysis

Total cell or tissue extracts were extracted in cell lysis buffer followed by immunoblotting with anti-ADAM19 (1:1000, Abcam, Cambridge, MA, USA), and anti-β-actin (1:2000, Santa Cruz Biotechnology, Santa Cruz, CA) as described previously [12].

### Luciferase reporter assay

To construct the pMIR-ADAM19-3’UTR plasmid that contained the potential binding sites of the ADAM19 3’-UTR downstream of the firefly luciferase gene, a 282 bp sequence was amplified and inserted into the SpeI and HindIII sites of the pMIR-REPORT Luciferase vector(Ambion, Austin, TX, USA). The plasmid with the miR-30c target site deleted from the ADAM19 3’-UTR was also constructed. HEK293 and HCT116 cells were used to measure luciferase activity. When grew to 60–70% confluence, cells were co-transfected with 100 ng Luciferase plasmid and 50 ng Renilla plasmid (Ambion, Austin, TX, USA) along with 650 ng miR-30c mimic or NC as described above. After incubation for 48h at 37°C,the luciferase activity was detected with the Dual Luciferase Reporter 1000 Assay System (Promega, Madison, WI, USA).

### In vivo tumor growth assays

Female athymic BALB/c nu/nu mice (aged 4 weeks) were purchased from Shanghai Laboratory Animal Center (Shanghai, China). All animal procedures were in accordance with Harbin Medical University Institutional Animal Care and Use Committee guidelines. The 2nd Affiliated Hospital of Harbin Medical University Animal Care and Use Committee approved this research. The animals were housed as described previously [12]. 5×10^6^ HCT116 cells that were stably transfected with miR-30c were injected subcutaneously to the right flank of nude mice. Tumor size was measured by caliper every 4 days. Both maximum (L) and minimum (W) length of the tumor were measured and the tumor size was calculated as ½LW^2^. After 30 days, the mice were sacrificed and photographed. Tumors were harvested and weighed. Additionally, 2×10^6^ cells were injected in the tail veins of nude mice. After 18 days, the lungs were removed and rinsed, and the pulmonary metastatic colonies were counted by visual inspection. All surgery was performed under sodium pentobarbital anesthesia, and all efforts were made to minimize suffering. Three animals were included in each group.

### Statistical analysis

The software SPSS V20.0 was used for statistical analysis. All values are expressed as mean ± SEM and all data has been repeated at least three times. Student’s t-tests were used to determine the statistical significance of differences between groups. Spearman’s correlation was applied to identify the correlation between miR-30c expression and ADAM19 expression. Differences with *p*<0.05 were considered significant.

## Results

### **MiR**-30c **was down-regulated in colon cancer tissues**

**Fig 1:**
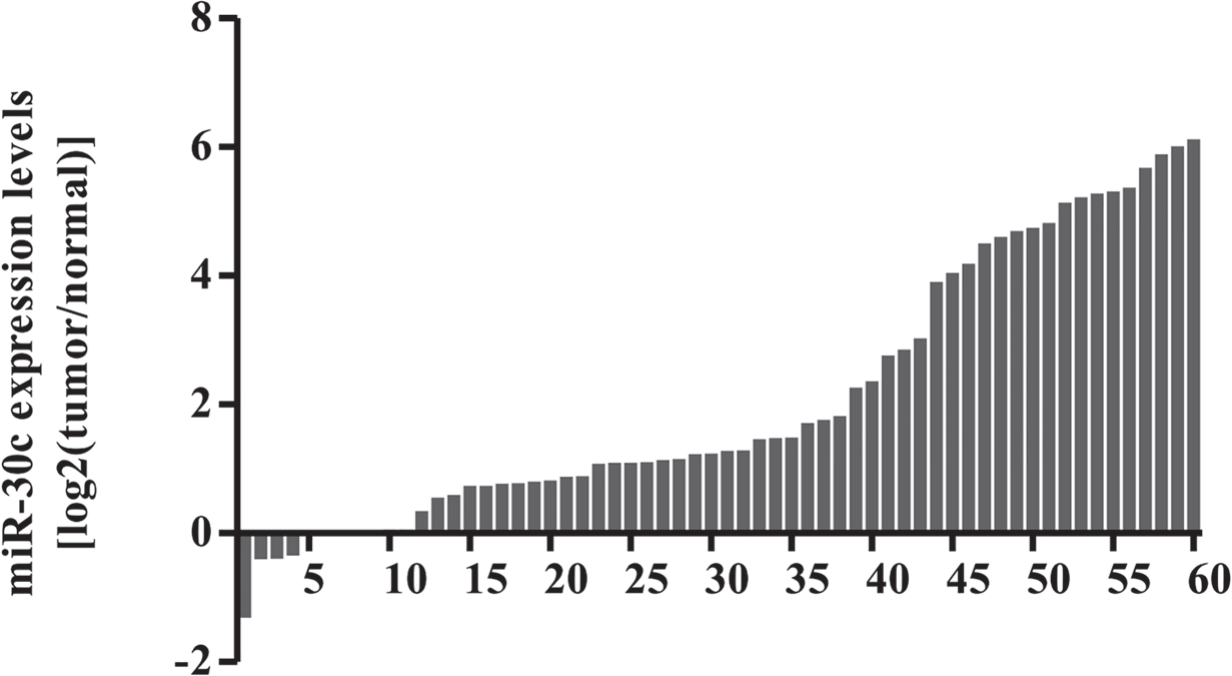
MiR-30c was down-regulated in colon cancer tissues. MiR-30c expression was examined by qRT-PCR in 60 pairs of human colon cancer tissues. 54 out of 60 tumor samples showed decreased expression of miR-30c compared with the adjacent normal mucosa tissues. MiR-30c expression was normalized to that of U6 in each sample.

To determine the expression of miR-30c in clinical tissues, we collected 60 pairs of human colon cancer samples and their adjacent, non-tumorous mucosa tissues. As indicated by the results of qRT-PCR analysis, miR-30c expression level was obviously down-regulated in 54 out of 60 tumor samples compared with the adjacent normal mucosa tissues (Fig. 1). The association between miR-30c and clinicopathological feature was analyzed (Table 2). These data indicate that miR-30c is decreased in colon cancer, and it may be correlated with human colon cancer progression.

**Table 2:** Characteristics and miR-30c expression in colon cancer patients.

Clinicopathological features	No. of patients (%)	miR-30c level (log2 transformed)mean ± s.d.	*P* value
Age			0.87
≤66	30(50.0)	2.14±1.90	
>66	30(50.0)	2.06±2.21	
Gender			0.39
Male	44(73.0)	2.24±2.10	
Female	16(27.0)	1.72±1.90	
Tumor size(cm^2^)			0.35
≤16	30(50.0)	1.85±1.98	
>16	30(50.0)	2.35±2.11	
Staging			0.324
I	4(6.7)	2.46±2.70	
II	18(30.0)	2.25±2.11	
III	32(53.3)	2.02±2.02	
IV	6(10.0)	1.83±2.06	
Differention level			0.15
well-moderately	6(10.0)	1.74±2.68	
moderately	36(60.0)	1.83±1.94	
moderately-poorly	16(26.7)	2.99±2.00	
poorly	2(3.3)	0.91±0.25	
CEA	/	/	0.04(R = -0.80)*
CA199	/	/	0.06(R = -0.66) *

* The correlation between miR-30c and CEA and CA199 is determined by Spearman’s correlation.

### **MiR**-30c **inhibited cell proliferation in vitro**

**Fig 2:**
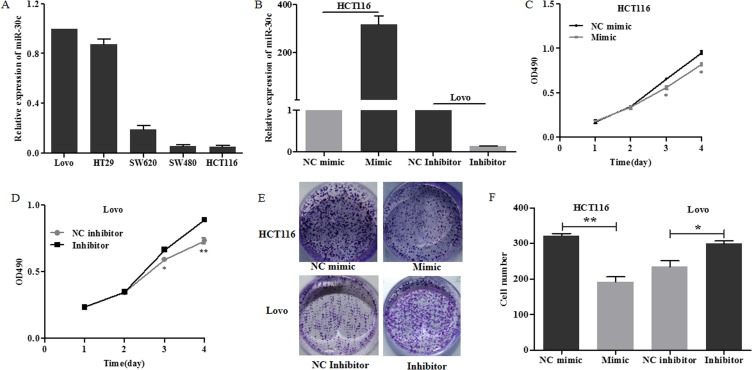
MiR-30c inhibited cell proliferation in vitro. (A) Relative expression levels of miR-30c in five colon cancer cell lines were detected with the quantitative real-time PCR (qRT-PCR). Columns, average of three independent experiments; bars, S.E. (B) HCT116 or Lovo cells were transiently transfected with miR-30c mimic or NC mimic and inhibitor or NC inhibitor respectively. The expression of miR-30c was determined by qRT-PCR after 24h. (C-D) Effects of miR-30c on the proliferation were examined by MTT assay. Points are the average of three independent experiments; bars represent S.E.; *p<0.05; ** *p*<0.01. (E) Effects of miR-30c on the proliferation of HCT116 cells were examined by colony formation assay. (F) The number of clones was quantitatively analyzed. *p<0.05; **p<0.01.

QRT-PCR was applied to detect relative expression level of miR-30c in different colon cancer cell lines and Lovo was used as the control group. As shown in Fig. 2A, HCT116 had a relative lower miR-30c expression level among these five cell lines. Therefore, we transiently transfected miR-30c mimics into HCT116 cells and miR-30c inhibitor into Lovo cells and miR-30c expression was validated by qRT-PCR (Fig. 2B). These cells were subsequently subjected to MTT assay. As expected, miR-30c inhibitor promoted while miR-30c mimic suppressed the proliferation of cells (Fig. 2C, 2D). To thoroughly investigate the phenotype, colony formation assay was adopted. Consistently, miR-30c overexpression suppressed the colony formation of colon cancer cells (Fig. 2E, 2F).

### **MiR**-30c **inhibited cell migration and invasion ability of colon cancer cells**

**Fig 3:**
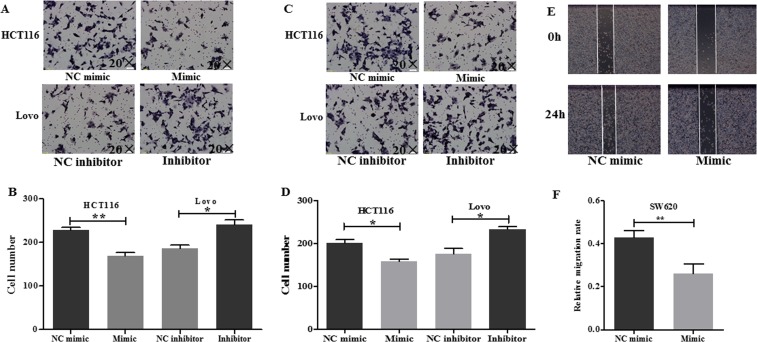
MiR-30c inhibited cell migration and invasion ability of colon cancer cells. (A-B) Effects of miR-30c on migration were analyzed by Transwell migration assay. A, representative photos; B, quantitative analysis. (C-D) Effects of miR-30c on invasion were analyzed by Transwell invasion assay. C, representative photos; D, quantitative analysis. (E-F) F, Wound healing assay was adopted to evaluate the effect of miR-30c on migration. The artificial gap was through the central axis when cells reached a density of 80%. Photos of cells were taken at 0 and 24 h. E, representative photos; F, quantitative analysis. *p<0.05; ** *p*<0.01.

To understand the impact of miR-30c on cell migration and invasion, Transwell migration and invasion and wound healing assays were performed. The results indicated that overexpression of miR-30c obviously inhibited while down-regulation of miR-30c promoted migration and invasion abilities of colon cancer cells (Fig. 3A-3D). Moreover, wound healing assay was applied to study the influence of miR-30c on migration of SW620 cells (Fig. 3E, 3F). The results showed that miR-30c could inhibit migration rate of colon cancer cells. Taken together, miR-30c was likely to play an important role in cell migration and invasion.

### ADAM19 was a direct target of miR-30c

**Fig 4:**
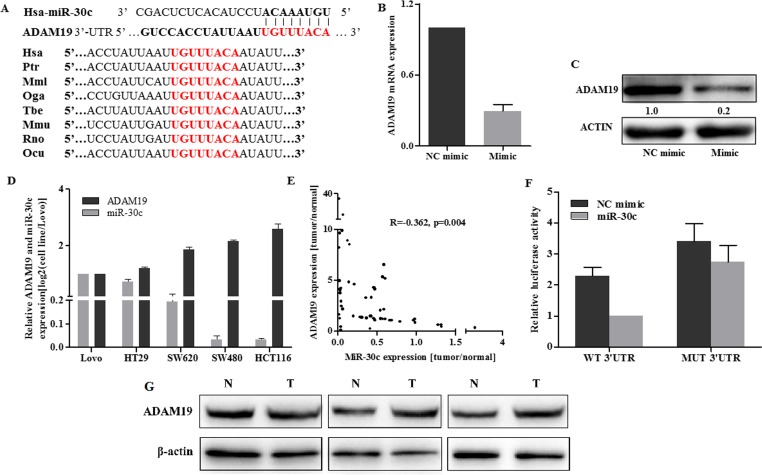
ADAM19 was a direct target of miR-30c. (A) The predicted targeting site with miR-30c of ADAM19 3’-UTR; MiR-30c targeting sequences of ADAM19 3’-UTR are evolutionarily conserved through eight species (Hsa: human; Ptr: chimpanzee; Mml: rhesus; Oga: bushbabay; Tbe: treeshrew; Mmu: mouse; Rno: rat; and Ocu: rabbit.). The targeting sites are highlighted in red. (B-C) qRT-PCR and western blot analysis was applied to detect mRNA and protein expression of ADAM19 in HCT116 cells after transfection of miR-30c mimic or NC mimic. (D) ADAM19 mRNA and miR-30c expression level was determined in five colon cancer cell lines by qRT-PCR. (E) ADAM19 mRNA and miR-30c levels were inversely correlated in colon cancer tissues as determined by qRT-PCR. β-actin and U6 were used as the endogenous controls, respectively. (F) HEK293T cells were co-transfected with wild-type or mutant reporters and the miR-30c mimic or negative control (NC mimic). After 48h, Luciferase/Renilla activity was measured. (G) ADAM19 was upregulated in colon cancer tissues compared with normal tissues, as indicated by Western blotting.

Bioinformatics strategies were used to search the potential targets of miR-30c that mediated miR-30c’s growth and metastasis inhibition. All of the four bioinformatics algorithms, miRBase, TargetScan, PicTar, and miRanda were used and then miR-Ontology Database was applied. Finally, ADAM19 was identified as a cancer-associated gene. ADAM19 3’-UTR possessed a perfect complementary region at position 3539–3546nt for miR-30c. In addition, TargetScan showed that the binding sites of miR-30c are evolutionarily conserved in various vertebral species (Fig. 4A). Furthermore, both western blot and qRT-PCR suggested a down-regulation of ADAM19 in HCT116 cells after transfection with miR-30c mimic (Fig. 4B, 4C). Further experiments demonstrated that the expression levels of miR-30c and ADAM19 were inversely correlated in several colon cancer cell lines and clinical tumor samples determined by qRT-PCR (Fig. 4D, 4E). Consitently, ADAM19 was upregulated in tumor tissues detected by western blot (Fig. 4G). These data proved that ADAM19 was likely to be targeted by miR-30c both transcriptionally and post-transcriptionally. To explain the detailed mechanism, luciferase reporter assay was performed. As shown in Fig. 4E, transient co-transfection of miR-30c mimic and the pmiRGLO-wt 3’-UTR vector (which contained the miR-30c target site) into HEK293T cells with led to a marked reduction in reporter activity. However, the reduction was abolished when miR-30c mimic and a construct in which the target site was deleted from the ADAM19 3’-UTR-reporter were transfected into HEK293T cells (Fig. 4F). Taken together, these findings revealed that miR-30c inhibited ADAM19 expression by directly targeting the ADAM19 3’-UTR.

### ADAM19 mediated tumor suppressive effect of miR-30c

**Fig 5:**
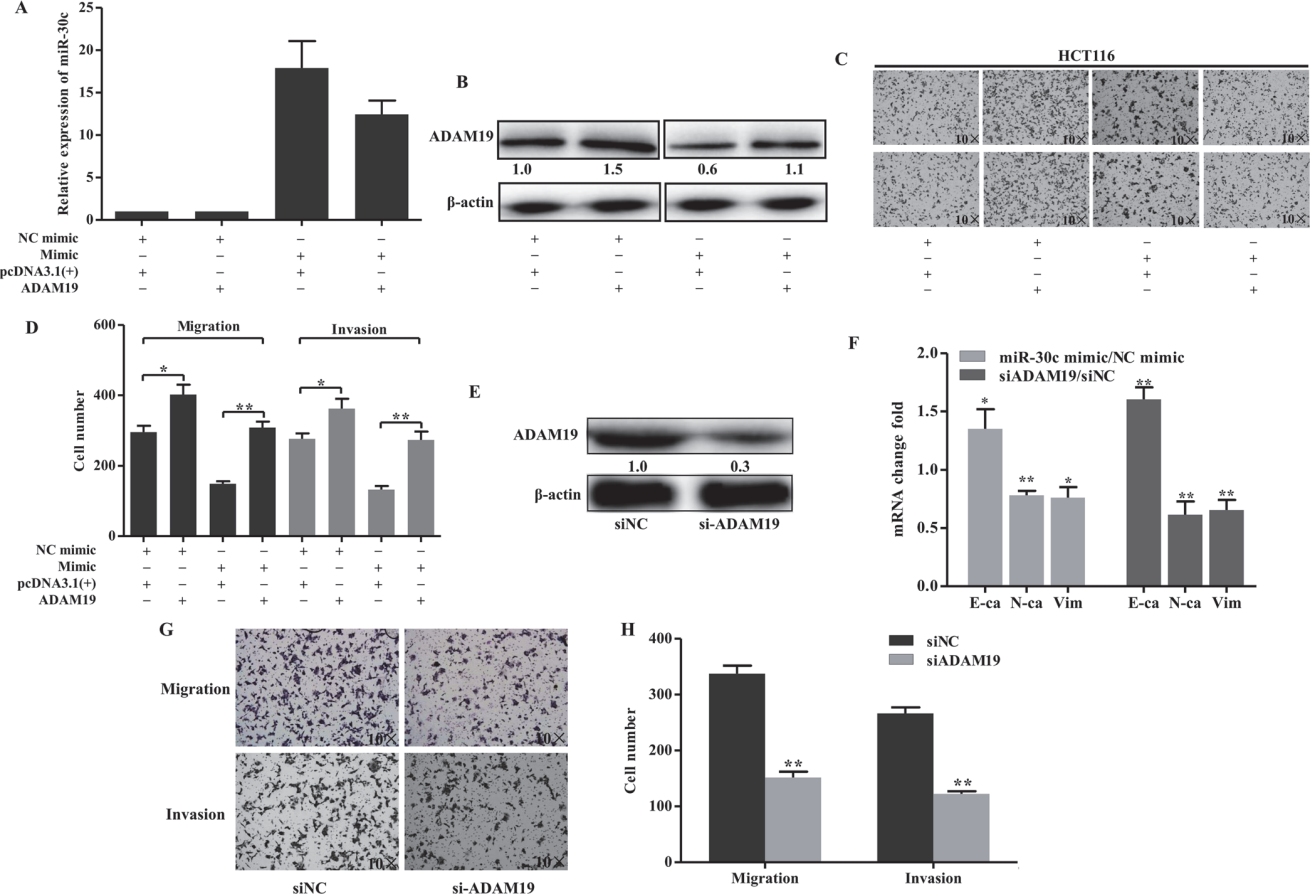
ADAM19 mediated tumor suppressive effect of miR-30c. (A-B) The expression of miR-30c and ADAM19 were examined by qRT-PCR (A) and western blot analysis(B), respectively in HCT116 cells which were co-transfected with miR-30c mimic (or NC mimic) and ADAM19 (or pcDNA3.1(+)).β-actin was used as the endogenous control. (C-D) The cells were subjected to Transwell migration and invasion assays. C, representative Transwell pictures; D, quantitative analysis. (E) HCT116 cells were transfected with ADAM19 siADAM19 or siRNA negative control (siNC). After 48h, ADAM19 expression was detected with western blot. (F) E-cadherin, N-cadherin and vimentin were detected by qRT-PCR after transfected with siADAM19/siNC and miR-30c mimic/NC mimic. The columns represented the relative expression to siNC and NC mimic groups respectively. (G-H) After transfected with siRNA or siNC, HCT116 cells were subjected to Transwell migration and invasion assays. G, representative Transwell pictures; H, quantitative analysis. *p<0.05; ** *p*<0.01.

Based on these above evidences, we made the hypothesis that ADAM19 attributed to the anti-tumorous effect of miR-30c. To prove this untested hypothesis, we constructed a vector using a cDNA that did not contain the 3’-UTR of ADAM19, afterwards the vector and miR-30c mimic/NC mimic were co-transfected into HCT116 cells. MiR-30c and ADAM19 expression were examined with qRT-PCR and western blot respectively (Fig. 5A, 5B). Transwell migration and invasion assays showed that restoration of ADAM19 was able to abolish miR-30c induced migration and invasion inhibition (Fig. 5C, 5D). Of interest, Transwell assays indicated that knockdown of ADAM19 by siRNA could suppress migration and invasion of cells, which was similar to the effect of miR-30c overexpression (Fig. 5E, 5G and 5H). In addition, we determined the effect of miR-30c and ADAM19 on EMT process. The qRT-PCR results demonstrateed that E-cadherin was upregulated while N-cadherin and vimentin were inhibited by overexpression of miR-30c or silencing of ADAM19 (Fig. 5F), therefore miR-30c and ADAM19 might be involved in the regulation of EMT process. Overall, ADAM19 might be a functional target of miR-30c.

### **MiR**-30c **inhibited tumor growth and metastasis in vivo**

**Fig 6:**
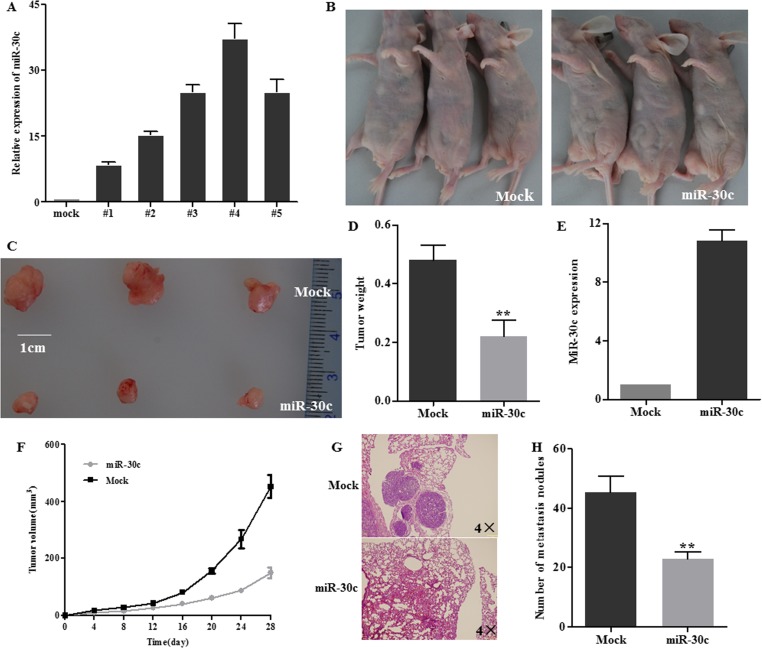
MiR-30c inhibited tumor growth in vivo. (A) The stable overexpression of miR-30c in HCT116 cell clones was determined with qRT-PCR. (B) 5×10^6^ HCT116 cells which were stably transfected with miR-30C or empty vector (mock) were subcutaneously injected into nude mice (n = 3). Mice were sacrificed 28 days after injection. (C) Tumors were harvested and representative tumors were shown. (D) Tumors were weighted and the miR-30C overexpression group had a lower weight compared with the mock group. (E) MiR-30c overexpression resulted in inhibition of growth rate. (F) The expression of miR-30c was detected with qRT-PCR in tumors. (G-H) Hematoxylin and eosin staining of sections of lung and statistical results of metastasis nodes. ** *p*<0.01.

To analyze the function of miR-30c in vivo, the nude mice xenograft model was used. We first constructed miR-30c stably overexpressing cells using HCT116 cell line (Fig. 6A). As it had the highest miR-30c expression, the #4 clone was selected in the following experiments. The cells were subcutaneously injected into flank of nude mice and tumor sizes were monitored every four days. Mice were sacrificed and subcutaneous tumors were weighted after four weeks. The results revealed that miR-30c led to a markedly decline in tumor size and weight compared with the mock group (Fig. 6B-6D). As shown in Fig. 6E, miR-30c decreased the growth rate of tumors in vivo. In addition, the expression of miR-30c in miRNA treated group was higher than that in mock group (Fig. 6F). What’s more, in vivo tumor metastasis assay showed that miR-30c could inhibit lung metastasis (Fig. 6G, 6H). Overall data suggested the anti-tumor effect of miR-30c in vivo.

## Discussion

MiRNAs are deregulated in various types of cancers and thus function as tumor-suppressors or oncogene by interaction with their corresponding targets. However, relatively limited information underlying the detailed mechanism of miRNA involvement in cancers is known. MiR-30c has been investigated in a number of cancers. It is down-regulated most of reported researches such as in non-small cell lung cancer, prostate cancer, leukemia, endometrial cancer, breast cancer and colorectal cancer [8,19,20,21,22]. However, several studies have reported the up-regulation of miR-30c in breast cancer, renal cell carcinoma [23,24]. MiR-30c exerts its function by involving in tumor progression, predicting prognosis or chemotherapy efficacy in these cancers. Of interest, some controversial evidences exist with regard to the function of miR-30c. This elucidates the complicated role of miR-30c in tumourigenesis and progression, despite its anti-tumor or oncogenic nature. In our study, we present that miR-30c might inversely regulate colon cancer progression by suppressing cell proliferation, migration and invasion. Moreover, miR-30c is decreased both in clinical specimens and cell lines along with up-regulated levels of ADAM19. ADAM19 is a directly functional target of miR-30c identified by luciferase assays and in vitro experiments. All our data suggests that miR-30c plays an important role in colon cancer.

To identify the potential targets of miR-30c, four bioinformatics algorithms were applied. After a primary screening, ADAM19 is identified as the target of miR-30c because it is closely associated with tumorous process. ADAM is an matrix metalloproteinase-related protein family which belongs to the zinc protease superfamily. The ADAM family could bind with integrin receptor and act as metalloprotease. They are also presented with a cytoplasmic domain [25]. The ADAMs (disintegrin metalloproteases)family are involved in a plethora of activities including membrane fusion, growth factor cytokine and shedding, cell migration, along with muscle development, fertilization, and cell fate determination [26]. ADAMs could degrade ECM molecules and promote cancer metastasis due to their proteinase activity which is similar to MMPs. There are more than twenty members of ADAM family. ADAM19 is an important member of ADAM family. Various studies have shown that ADAM19 is involved in many types of diseases. It is reported that possibility that mutations in ADAM19 may contribute to human congenital heart valve and septal defects[27]. Some researches from China showed that ADAM19 might have effects on testis development and chronic obstructive pulmonary disease. including cancer. What’s more, some data suggest that ADAM19 may be involved in the key processes of glandular secretion, trophoblast invasion and degradation of extracellular matrix during early pregnancy[28]. In addition, ADAM19 was found to be up-regulated in renal cell carcinoma and primary brain tumors and promoted the invasiveness of the tumors[29,30]. There are relatively little studies on miRNA targeting ADAMs despite that a recent study found transforming growth factor-β signaling regulates ADAM expression in experimental renal fibrosis via miR-29 [31]. The function of ADAM19 still remains unclear. In our study, we found that ADAM19 could promote the invasiveness of colon cancer cells for the first time.

MiR-30c belongs to the miR-30 family which consists of five members: miR-30a, b, c, d, and e. Interestingly, these five members have different functions. MiR-30c has been shown to function as either oncogene or tumor suppressor in different types of cancer. Consistently, miR-30a acts as a tumor suppressor in considerable types of malignant tumors, including lung cancer, breast cancer, gastric cancer, thyroid cancer, and leukemia [6,32,33,34,35]. MiR-30b could prohibit apoptosis of glioma cells and lung cancer cells [36]. Whereas, miR-30b is capable of reducing cell growth in breast cancer and prevent EMT in liver cancer with interaction with cyclin E2 (CCNE2) and Snail1, respectively [32,37]. However, miR-30d is likely to function as an oncogene in a majority of researches [38,39,40,41]. Similarly with miR-30a, miR-30e might inhibit proliferation in cancers [16] and is also recognized as a prognosis predictor in nasopharyngeal carcinoma [42]. This phenomenon is reasonable as miRNA perform in targets-dependent and tissue-specific way. The miRNA-30 family share the same seed sequence, however, no dramatic additional effects was generated after the enforced expression of these five members simultaneously (data not shown) which suggests there is no synergetic impact of them. The inhibition effect of miR-30c on ADAM19 is most obvious among all the five members of miR-30 family indicated by qRT-PCR and Luciferase assay. Therefore we focus on miR-30c in our study.

It is well known that one single miRNA could regulate multiple targets and each mRNA could also be controlled by several miRNAs. In our study, we found that the miR-30c induced a 20% reduction of ADAM19 expression, however, the migration and invasion rate increased more than 20% (Fig. 5B VS 5D). It indicated that some other gene Targets are involved in changes induced by miR-30c.

Taken together, the present study shows for the first time that miR-30c suppresses cancer cell growth, migration and invasion by directly targeting ADAM19 which could promote the malignance of colon cancer cells. The newly identified miR-30c/ADAM19 axis shed new light on the miRNA-based regulatory mechanism. Considering the crucial role of miR-30c in colon cancer, manipulation of miR-30c may represent a potential novel therapeutic target for treating colon cancer.
